# Chromosome mapping in *Abracris flavolineata* (De Geer, 1773) (Orthoptera) from the Iguaçu National Park – Foz do Iguaçu, Paraná, Brazil

**DOI:** 10.3897/CompCytogen.v11i2.10282

**Published:** 2017-03-31

**Authors:** Mariana Bozina Pine, Raquel Bozini Gallo, Carlos Roberto Maximiano da Silva, Larissa Forim Pezenti, Fernando Campos De Domenico, Vilma Loreto, Renata da Rosa

**Affiliations:** 1 Departamento de Biologia Geral, CCB, Universidade Estadual de Londrina (UEL), Londrina, Paraná, Brazil; 2 Museu de Zoologia, Instituto de Biociências, Universidade de São Paulo (USP), São Paulo, São Paulo, Brazil; 3 Departamento de Genética, CCB, Universidade Federal de Pernambuco, Recife, Brazil

**Keywords:** Acrididae, Brazilian Atlantic forest, chromosome banding, fluorescence *in situ* hybridization, grasshopper

## Abstract

In this paper, we present the cytomolecular analysis of a population of *Abracris flavolineata* collected in the largest fragment of the Brazilian Atlantic forest, the Iguaçu National Park. The diploid number in males was 23 (22+X0), with two large pairs (1–2), 7 medium (3–9), 2 small (10–11) and the X chromosome of medium size. Heterochromatic blocks were evident in the pericentromeric regions of all chromosomes. Heterogeneity in the distribution of heterochromatin was observed, with a predominance of DAPI^+^ blocks. However, some chromosomes showed CMA_3_^+^ blocks and other DAPI^+^/CMA_3_^+^ blocks. The 18S rDNA sites were distributed on the short arms of 5 pairs. In two of these pairs, such sites were in the same chromosome bearing 5S rDNA, and one of the bivalents, they were co-located. Histone H3 genes were found on one bivalent. The results added to the existing cytogenetic studies provided evidence of great karyotypic plasticity in the species. This pliancy may be the result of vicariant events related to the geographical distribution of different populations of *A. flavolineata*.

## Introduction

The family Acrididae is one of the most speciose, heterogeneous and conceivably the most recent in the Acridoidea group (Song et al. 2015). Among its subfamilies, Ommatolampidinae comprises 9 tribes and over 50 genera, with geographical distribution in North, Central and South America (Amédégnato 1974, Carbonell 1977). This subfamily is associated with secondary growth of vegetation in dry forest areas (Amédégnato and Decamps 1980, Rowell 1987), or even in clearings within Tropical rainforests (Braker 1991, Sperber 1996). The genus *Abracris* Walker, 1870 belongs to this subfamily and is distributed in the Neotropical region, from Mexico to Argentina (Roberts and Carbonell 1981).

Although there are several cytogenetic studies of Neotropical Acrididae species, especially of the subfamily Ommatolampidinae, there are few studies that elucidate the molecular structure of chromosomes. Specific C-banding techniques for the identification and classification of chromosomes have been applied more often in *Abracris flavolineata* species (De Geer, 1773) (Cella and Ferreira 1991, Rocha et al. 2011).

According to Cella and Ferreira (1991), *Abracris flavolineata* shows 2n=24/23 (females/males), with the XX/X0 sex chromosome system. Seven subtelocentric, two metacentric and two submetacentric pairs, and the subtelocentric chromosome X make up the karyotype of *A. flavolineata*. In addition, this species displayed B chromosomes (Cella and Ferreira 1991, Bueno et al. 2013). Molecular markers were applied to understand their molecular composition and mechanisms of evolution (Bueno et al. 2013, Milani and Cabral-de-Mello 2014, Palacios-Gimenez et al. 2014).

Knowledge of Ommatolampidinae is limited, considering the diversity of the subfamily. Few cytogenetic tools have been applied, and the only samples that have been analyzed were from southeastern Brazil (Rio Claro/São Paulo state). To improve the knowledge about this species, in particular about this group, it was necessary, not only to use more cytogenetic tools but also study samples from other regions of Brazil. Thus, this work aimed to study specimens of *A. flavolineata* collected in the Iguaçu National Park (Southern Brazil), a paramount area of the Brazilian Atlantic, using different cytogenetic markers in order to understand the evolutionary mechanisms present in this group of insects.

## Material and methods

The current study used twenty male specimens of *Abracris flavolineata* collected from the Iguaçu National Park, Foz do Iguaçu, Paraná State, Brazil – 25°37'40.67"S; 54°27'45.29"W (DDM). The individuals were identified and deposited in the Museu de Zoologia da Universidade de São Paulo (MZUSP) and collected with the permission of Instituto Chico Mendes de Conservação da Biodiversidade – ICMBio, protocol number 31946-2. The insects were anesthetized and dissected before ﬁxing their testes in methanol: acetic acid (3:1). Chromosome preparations for mitotic and meiotic analyses were made through cell suspension by maceration in one drop of 45% acetic acid. Heterochromatin distribution was analyzed by Giemsa C-banding (Sumner 1972). The GC- and AT-rich bands were detected with chromomycin A_3_ (CMA_3_) and 4’-6-diamino-2-phenylindole (DAPI), respectively (Schweizer et al. 1983).

In addition to the karyotype studies, genomic DNA was extracted from the muscle tissue of a male specimen using the phenol/chloroform procedure described by Sambrook and Russel (2001). Unlabelled 18S rDNA, 5S rDNA and Histone H3 gene probes were generated by polymerase chain reaction (PCR) using the primers: 18S rDNAF 5‘-CCTG AGAAACGGCTACCACATC-3’ and 18S rDNAR 5‘-GAGTCTCGTTCGTTATCGGA-3’(Whiting 2002); 5SrDNAF 5’-AACGACCATACCACGCTGAA-3’ and 5SrDNAR 5’-AA GCGGTCCCCCATCTAAGT-3’ (Loreto et al. 2008a); H3F 5’-ATATCCTTRGGCATRAT RGTGAC-3’ and H3R 5’-ATGGCTCGTACCAAGCAGACVGC-3’ (Colgan et al. 1998). The probes isolated by PCR were labeled with digoxigenin-11-dUTP and biotin-11-dATP by PCR. The *in situ* hybridization procedure was performed according to Pinkel et al. (1986).

## Results and discussion

**Figure 1.**
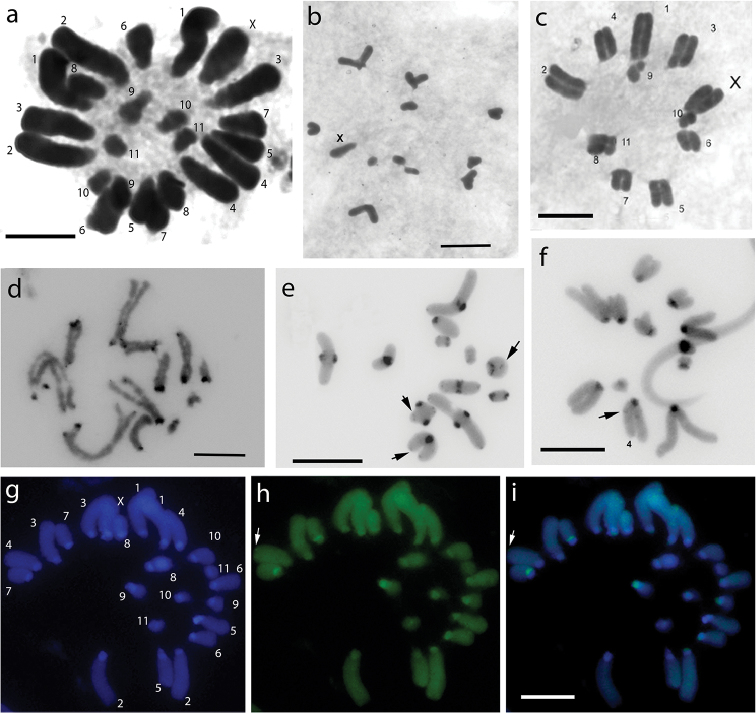
Mitotic and meiotic stages of *Abracris flavolineata* by Giemsa conventional staining (**a–c**), C-banding (**d–f**) and fluorochromes staining (**g–i**): **a** mitotic metaphase **b** diakinesis **c** metaphase II **d** pachytene **e** diakinesis **f** metaphase II **g** mitotic metaphase by DAPI staining **h** mitotic metaphase by CMA_3_ staining **i** overlapping DAPI/CMA_3_. The numbers correspond to autosomes. X corresponds to the sex chromosome. The arrows indicate the discrete heterochromatic bands. Bar= 5µm.

All samples of *Abracris flavolineata* collected in the Iguaçu National Park presented 2n=23 (♂) and an XX/X0 sex-determination system (Fig. 1). The chromosomes were classified into two large pairs (1–2), 7 medium pairs (3–9) and 2 small pairs (10–11). The X chromosome is medium-sized. Pairs 1 to 7 and the X chromosomes are subtelo-acrocentric; pairs 8 and 9 are submetacentric, and pairs 10 and 11 are metacentric (Fig. 1a). The X chromosome presented itself univalent at diakinesis (Fig. 1b).

The karyotype structure observed in this study is conserved regarding the diploid number and morphology of the A chromosomes, corroborating the data already established for the species by Cella and Ferreira (1991) and by Bueno et al. (2013) studying specimens from Rio Claro (southeastern Brazil). However, these authors reported the occurrence of supernumerary chromosomes, which were not found in samples from National Iguaçu Park. The sex-determination pattern considered the ancestor of grasshoppers was present in specimens of *Abracris flavolineata*, with males with the X0 and females with the XX sex chromosome system. In Acrididae, some species, such as *Rhammatocerus brasiliensis* (Bruner, 1904), *R. brunneri* (Giglio-Tos, 1895), *R. palustris* Carbonell, 1988, *R. pictus* (Bruner, 1900), *Orthoscapheus rufipes* (Thunberg, 1824), among others, show a karyotype similar to that of *Abracris flavolineata*, with 2n = 23,X0 and 24,XX, being considered the ancestral pattern of the family (White 1973; Loreto et al. 2008b; Rocha et al. 2011).

Heterochromatic blocks were shown in the pericentromeric regions of all chromosomes (Fig. 1d–i). Chromosome 4 showed a slight heterochromatic block in the proximal region of the long arm (Fig. 1f). Nevertheless, this block was also viewed in most meiotic stages. It was possible note discrete heterochromatic bands in some chromosomes (Fig. 1d–f). These bands appear in some cells and not others, and this may be the result of differences in the condensation of chromosomes in different cells or a technical artifact. The position of heterochromatin at pericentromeric regions is a common feature in Acrididae, being observed in several species (Loreto and Souza 2000, Rocha et al. 2004). The heterochromatin plays a very important role in the evolution of karyotypes. The pericentromeric preferential distribution may be related to equilocal transfer between non-homologous chromosomes of similar size by positioning in the nucleus by bouquet configuration (Schweizer and Loidl 1987).

Staining with fluorochromes CMA_3_/DAPI showed heterogeneity in the distribution of heterochromatin on autosomes: (i) DAPI^+^ bands located at pericentromeric regions in most chromosomes; (ii) pericentromeric DAPI^+^/CMA_3_^+^ bands on 2 and 6 chromosomes; (iii) a discrete CMA_3_^+^ band in the terminal region on chromosome 4; and (iv) DAPI^+^ adjacent to CMA_3_^+^ blocks on three pairs (5, 7 and 9). The X chromosome showed adjacency of DAPI^+^-CMA_3_^+^-DAPI^+^ bands (Figure 1g-i). The distribution of these blocks can be best demonstrated in the ideogram (Figure 3).

There are three patterns of distribution of GC-rich blocks in grasshoppers: (i) CMA_3_^+^ bands related to NORs location (Souza et al. 1998, Loreto and Souza 2000, Rocha et al. 2012); (ii) CMA_3_^+^ in all chromosomes (Souza et al. 1998, Pereira and Souza 2000); and, (iii) GC-rich blocks in some chromosomes (Loreto and Souza 2000, Souza et al. 2003, Loreto et al. 2005, Souza and Melo 2007, Rocha et al. 2012). AT-rich heterochromatin is rarely encountered in Acrididae, as is the case *Arcyptera fusca* (Pallas, 1773), *A. tornosi* Bolívar, 1884 (Bella and Gosálvez 1991) and *Dociostaurus genei* (Ocskay, 1832) (Rodríguez Iñigo et al. 1993). In *Abracris flavolineata*, studies show that most heterochromatin is GC-rich and there are no AT-rich blocks (Bueno et al. 2013). Thus, the pattern observed in specimens of *A. flavolineata* in this study shows a considerable karyotypic differentiation, where heterochromatinization processes, such as heterochromatin spreading, can be responsible for the differentiation of the Iguaçu National Park population, as in the grasshopper of the genus *Tropidacris* Scudder, 1869 (Rocha et al. 2015). Moreover, this differentiation of heterochromatin may be related to action of transposable elements (Grewal and Jia 2007).

**Figure 2.**
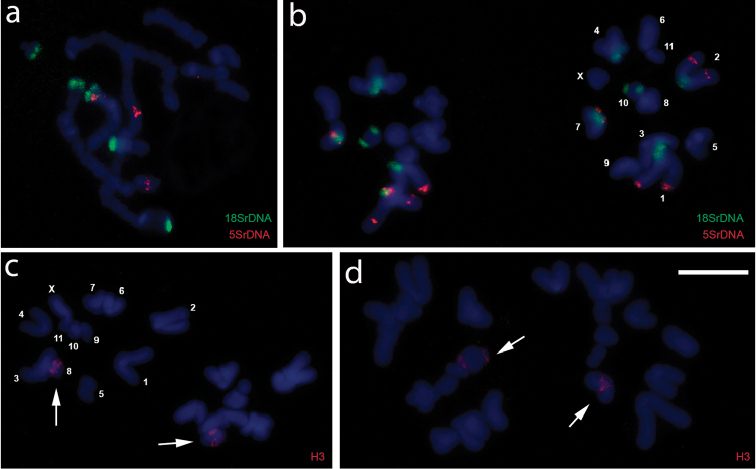
Fluorescent *in situ* hybridization of *A. flavolineata* in meiocytes: **a, b** 18S rDNA (green) and 5S rDNA (red) probes **c, d** histone H3 gene probe: **a** pachytene **b–d** two cells in metaphase II. The numbers correspond to autosomes. X corresponds to the sex chromosome. The arrows indicate the histone H3 gene sites. Bar= 5µm.

Although the position of rDNA sites was similar to that previously reported for other populations of this species, on terminal region, the number of sites was different from that observed in previous studies. The 18S rDNA sites were distributed on terminal regions of the short arms of all analyzed specimens of 5 pairs (1, 2, 4, 7 and 10), with no variations. Two of these sites were on the same chromosome as 5S rDNA sites. These sites were co-located on one of the bivalents (Fig. 2a and b). Bueno et al. (2013) reported a variation from 5 to 9 chromosomes bearing 18S rDNA in the species of the southeastern population. Conversely, the 5S rDNA sites were distributed in the terminal portion of the bivalent 1, in the interstitial region of bivalent 2 and pericentromeric region of 7. This pattern was more conservative where two sites (1 and 2) were similar to those observed by Bueno et al. (2013). A difference in the present study was the co-location of 18S rDNA and 5S sites on pair 7, where these sequences are located in heterochromatic regions. Cabral-de-Mello et al. (2011a) analyzed the distribution of 45S and 5S rDNA in 29 species of family Acrididae, and noted the predominance of the location of sites on separate chromosomes (80.3% of the clusters). Considering this study, the co-location of DNA sites may be associated with recent evolutionary processes in the species from the Iguaçu National Park.

**Figure 3.**
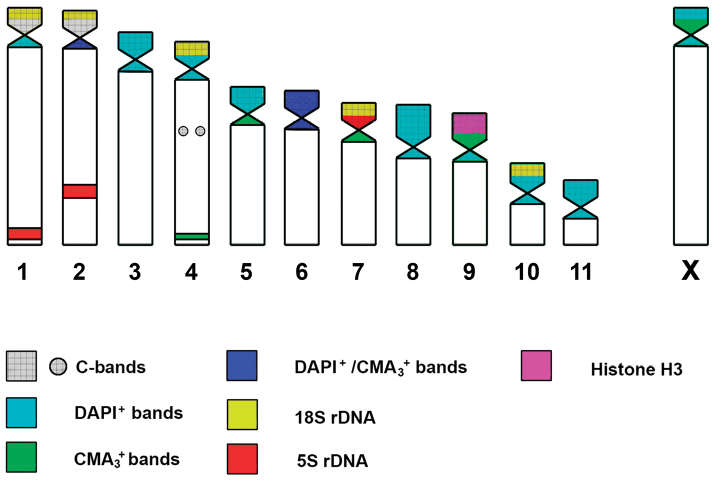
Idiogram showing the mapping of different sequences studied in *Abracris flavolineata* from the Iguaçu National Park.

Histone H3 genes were located on a corresponding bivalent of pair 8 (Fig. 2c and d). This location is similar in number to that found in most species of grasshoppers, distributed on a single pair of chromosomes (Cabrero et al. 2009, Cabral-de-Mello 2011a, 2011b, Neto et al. 2013). However, our data differ from those reported for the population of *A. flavolineata* of southeastern Brazil, in which these genes were observed on all chromosomes, including the X chromosome, but not on B chromosomes (Bueno et al. 2013). As in our results, the distribution of the histone gene was adjacent to the CMA_3_^+^ and DAPI^+^ heterochromatic blocks (Fig. 3), these genes are probably intercalated to heterochromatin blocks. Thus, the repetitive DNA present in the heterochromatin may play an important role in the dispersion of this sequence leading to differences in distribution between different histone genes of *A. flavolineata* populations studied herein.

Although the chromosome number and karyotype formula of the specimens studied here were the same as those described in the southeastern population (Cella and Ferreira 1991, Bueno et al. 2013), a considerable variation in the distribution of AT and GC-rich bands and specific gene sequences (18S, 5S and H3) was found. This variation refers to significant chromosomal changes, as spreading of heterochromatin can be involved in the evolution of this species. The populations studied by Cella and Ferreira (1991) and Bueno et al. (2013) inhabit Atlantic rainforest fragments, which once formed a continuous biome. The forest fragments mentioned above are approximately 790 km away from the population studied here (Iguaçu National Park). Such distance, coupled with the fact that these species probably underwent vicariance events that led to the disruption of these populations, caused reproductive isolation that led to chromosomal differences. The data obtained, together with the existing cytogenetic studies, allow us to suggest that *Abracris flavolineata* has an extensive karyotype plasticity.
